# Shift working and risk of lipid disorders: A cross-sectional study

**DOI:** 10.1186/1476-511X-5-9

**Published:** 2006-04-10

**Authors:** Masoumeh Ghiasvand, Ramin Heshmat, Reza Golpira, Vahid Haghpanah, Ali Soleimani, Payman Shoushtarizadeh, Seyed Mohammad Tavangar, Bagher Larijani

**Affiliations:** 1 Artesh University of Medical Sciences, Occupational Medicine Department, Iran; 2 Endocrinology and Metabolism Research Center (EMRC), Tehran Medical University of Medical Sciences, Iran; 3 Rajae Hospital Cardiovascular Research Center, Iran University of Medical Sciences, Iran; 4 Department of Pathology, Tehran Medical University of Medical Sciences, Iran

## Abstract

**Background:**

previous studies have indicated on association between shift work and lipid profile disturbances. Lipid profile disturbances could be due to internal desynchronization. The aim of this study was to analyze whether there is relationship between shift work and serum lipids, fasting blood glucose and hypertension.

**Results:**

A total of 424 rail road workers between the ages of 21 and 64 years in this study filled out a questionnaire, and total cholesterol, triglyceride and HDL-C concentration were measured after 12-hours fasting. Association between shift work and biochemical variables and blood pressure were measured. The X^2 ^and fisher's exact test was used for comparing the qualitative variables and for quantitative variables with normal distribution we used the parametric tests. Odds ratio (OR) with the 95% confidence interval (95% CI) was used for comparing the proportions of risk variables.

Sub-populations in this study were consisting of 158 (37.3%) shift workers and 266 (62.7%) day workers. High levels of total cholesterol (> 200 mg/dl) and LDL-cholesterol (> 130 mg/dl) were significantly more prevalent in nearly all groups of shift workers irrespective of age. But there is no differences in the serum levels of triglyceride, HDL-C, fasting blood glucose and blood pressure between shift workers and day workers.

Adjusted Odd's ratio for the effect of shift working on high serum total cholesterol and LDL-C level were 2.11(95%CI: 1.33–3.36) and 1.76(95%CI: 1.09–2.83), respectively.

**Conclusion:**

This study showed that high serum total cholesterol and LDL-C level were more common in shift workers than in day workers. This finding persisted after adjustment was made for age and food type. But there was no difference in the prevalence of HDL-C, triglyceride, fasting blood glucose and hypertension between shift working and day working. It was concluded that shift work is a risk factor for lipid profile disturbances.

## Background

Broadly defined, shift work involves work at times other than normal daylight hours of approximately 7:00 A.M, to 6:00 P.M. because 24-hours operations are an inevitable component of numerous industries, night work or shift work is a necessary condition of employment for a significant segment of the work force. Critical 24-hours operations include police and fire protection, medical care, transportation, communication, and energy and water utilities. Other industries require continuous processing or operate around the clock to optimize capital investment in machinery and other production materials.

Estimates of the number of person doing shift work range from 10% to 25% of all those employed. Almost all occupations or industries have employees engaged in shift work

Because of the necessity of 24-hours operations, shift workers often live at variance with the conventional pattern of human activity, which is highest in the day and evening hours. These deviations from the daytime (or diurnal) activity pattern place the shift worker in opposition to many human functions that oscillate within a 24-hours period. Physiologic process (e.g. metabolic rate), psychological process (e.g. short term memory), and social process (e.g. family interaction) all have demonstrated rhythmic increases and decreases in daily activity. These patterns are called circadian rhythm because they cycle about once a day. When working at night and sleeping during day these circadian rhythms move about even after weeks of night work no complete adjustment of the rhythms are made. The single rhythm moves at its own pace and several rhythms may come in disharmony with each other or the surrounding. This is labeled internal desynchronizatin [1].

Also lipids have a circadian rhythm. And in day orientation the circadian variation (given as percentage of the total variation) was 5.6% for HDL/total cholesterol ratio, 30.5% and 31.6% for HDL and total cholesterol, 33.5% for LDL and 38.5% for triglycerides[2]. These circadian rhythms follow a primary andogenic circadian rhythm [3], with triglycerides having the acrophase early the morning, the other later in the day.

There is also a yearly rhythmicity, cholesterol and LDL-cholesterol being up to 3–5% higher in winter (cold period), while HDL and triglycerides are modestly changed [4].

Working irregular hours, including night work and shift work, has been found to be associated with higher levels of lipid [5,6,18]. In a cross over design, Orth-Gomer followed 45 male police officers. Half of the men were first followed in counter clock wise rotation for four weeks. The other group worked vice-versa. There was a change in triglycerides so that values changed toward lower values in the clockwise compared to the middle of period [7].

The aim of the present epidemiological study was to investigate whether shift work is associated with lipid disturbances, fasting blood sugar and hypertension.

## Results

The mean age of the shift workers was 46.40 ± 7.68 years old and day workers was 38.69 ± 9.75 years old.

The mean duration of job experience of the shift workers was 21.13 ± 7.42 years and day workers was 14.53 ± 9.21 years. Table 1 shows the result of mean of variables in present study.

**Table 1:** Serum FBS, CHOL, LDL-C, HDL-C, TG levels, BP, BMI in shift workers and day workers

Parameters	Shift workers		Day workers		P-value
		
	Mean	± SD	Mean	± SD	
FBS (mg/dl)	91.24	± 25.50	88.50	± 21.43	0.238
Total CHOL (mg/dl)	196.69	± 36.60	182.92	± 37.46	0.000
TG (mg/dl)	176.12	± 90.77	178.55	± 79.62	0.773
LDL-C (mg/dl)	119.27	± 32.86	110.80	± 30.15	0.007
HDL- C (mg/dl)	47.29	± 14.12	52.29	± 22.68	0.013
SBP (mmHg)	119.81	± 11.90	114.86	± 16.02	0.000
DBP (mmHg)	76.81	± 7.92	74.25	± 10.19	0.004
BMI	25.55	± 3.43	25.56	± 3.74	0.977

P-Value: P < 0.05, SBP: systolic blood pressure, DBP: diastolic blood pressure, BMI: body mass index, CHOL: cholesterol, HDL-C: high density lipoprotein-cholesterol, LDL-C: low density lipoprotein, cholesterol, TG: triglyceride

The total cholesterol > 200 mg/dl and LDL-C> 130 mg/dl were more prevalent in shift workers (total cholesterol: 72.2%, LDL-C: 37.3%) than day workers (total cholesterol: 50.8%, LDL-C: 25.4%).

The prevalence of Fasting blood glucose ≥126 mg/dl was 3.2% in shift workers and 3% in day workers and for HDL-C< 45 mg/dl, Triglyceride> 200 mg/dl, Hypertension ≥140/90 mmHg and Body mass index (BMI) ≥30 in shift workers were 53.8%, 49.4%, 17.1%, 39.9% and in day workers were 60.6%, 49.2%, 11.7%, 41.3%.

Table 2 shows the results of prevalence of variable in work groups (shift and day workers)

**Table 2:** Prevalence and comparison of parameters between shift workers and day workers

Parameters	Shift Workers		Day Workers		P-value
		
	Number	(%)	Number	(%)	
FBS ≥126 mg/dl	5	(3.2)	8	(3)	0.938
Total CHOL > 200 mg/dl	114	(72.2)	134	(50.8)	0.000
TG > 200 mg/dl	78	(49.4)	130	(49.2)	0.980
LDL-C > 130 mg/dl	59	(37.3)	67	(25.4)	0.009
HDL-C < 45 mg/dl	85	(53.8)	160	(60.6)	0.170
BP ≥140/90 mmHg	27	(17.1)	31	(11.7)	0.123
BMI ≥30	63	(39.9)	109	(41.3)	0.775

P-Value: P < 0.05

The results of relative risk were crude OR: 2.51 (95% CI: 1.64–3.83) and Adjusted OR: 2.11 (95% CI: 1.33–3.36) for total Cholesterol, crude OR: 1.75 (95% CI: 1.14–2.68) and Adjusted OR: 1.76 (95% CI: 1.09–2.83) for LDL-C, crude OR: 0.75 (95% CI: 0.50–1.12) and Adjusted OR: 0.91 (95% CI: 0.58–1.42) for HDL-C, crude OR: 1.00 (95% CI: 0.67–1.49) and Adjusted OR:1.25 (95% CI: 0.80–1.95) for Triglyceride, crude OR: 1.04 (95% CI: 0.33–3.25) and Adjusted OR: 1.2 (95% CI: 0.33–4.32) for Fasting blood glucose, crude OR: 1.41 (95% CI: 0.80–2.50) and Adjusted OR: 0.73 (95% CI: 0.38–1.39) for Hypertension.

## Discussion

This study showed that high serum total cholesterol and LDL-C level were more common in shift workers than in day workers. This finding persisted after adjustment was made for age and food type. Our results are in agreement with the results in previous studies [12]. But other studies have been shown that there was no difference in serum total cholesterol and LDL-C levels between shift workers and day workers (11.9).

Lennernas et al documented that dietary intake is lower during night shift than during morning and afternoon shifts. According to them, the redistribution of food intake from diurnal eating to nocturnal eating is related to serum total cholesterol, LDL- cholesterol. Even if the dietary intake and quality are similar in day workers as well as shift workers, there are still differences in eating habits that might contribute to differences in levels of serum lipids [12].

We found no differences in the prevalence of hypertriglyceridemia when comparing shift workers and day workers. But previous studies have been shown high serum triglyceride levels to be more prevalent among shift workers than the day workers [7,9-11,14,17].

In our study, there was no difference in the prevalence HDL-cholesterol level between day workers and shift workers, so this results in agreement with the results in previous study [9,11]. But other studies have been shown low HDL-cholesterol serum level to be more prevalent between the shift workers than the day workers [13,15].

We found no difference in the prevalence of hypertension when comparing shift workers and day workers. These results are consistent with previous study [11]. But one study has been shown hypertension to be more prevalent between the shift workers than the day workers [16].

We didn't find difference in the prevalence of hyperglycemia between shift and day workers, so this result in agreement with the result in previous study [14].

On the basis of study by our group and other, it is recommended that in every work place where shift work is mandatory, a chronoclinic should be established. Trained health care personnel of the chronoclinic should monitor intermittently (preferably every alternate year) the state of the biological clock (synchronized or desynchronized?) of each shift worker. Upon discovering rhythm desynchronization his/her transfer from shift work to day work for at least one year should be recommended to the employer/management. This would perhaps real out the possibilities of ill-effects of shift work that are expected to be impinged upon the workers.

It has been proposed that while examining tolerance/in tolerance of a shift worker to rotational shift work, the levels of anxiety and mental health status of the individual under careful and thorough examination should be taken into consideration. Sleep-wake disorder is another important variable that cannot be simply ignored while ascertaining intolerance to shift work.

In addition appropriate chronotherapy should also be administered in to intolerant shift workers while they are being transferred from shift duty to day duty.

All these counter-measures, either individually or in combination may improve the coping ability of shift workers thus minimizing the occupational health hazards and maximizing their performance. This would substantially increase the productivity of the organization for which they are working.

## Methods

Among a cross-sectional study, a total of 424 male railroad workers filled out a questionnaire with questions about working condition, smoking habits, diet, level of physical activity, and family history of hypertension, diabetes, hyperlipidemia, and hypothyroidism. These subjects were all workers of repair workshops.

Shift workers were defined as work at times other than normal daylight hours of approximately 7:00 AM to 6:00 PM or work during the weekends. Blood pressure was measured in the sitting position after 5 minutes rest. Hypertension was defined as having a systolic blood pressure of 140 mmHg or more, or a diastolic blood pressure of 90 mmHg or more and answering yes about being on antihypertensive medication. Body weight was measured in light indoor clothing and recorded to the nearest Kg. height was measured to the nearest centimeter without shoes. Body mass index (BMI) was calculate as weight (Kg) divided by height squared (m^2^). Those with a BMI of 30 or more were classified as obese.

Total cholesterol, triglycerides and HDL-cholesterol concentration were measured in the after 12-hours fasting. Serum triglycerides, cholesterol and FBS levels were measured by enzymatic assays, HDL and LDL by direct immuno turbidometry assays (Pars-Azmun, Tehran, Iran). Serum samples from cases and controls were always analyzed in the same run. The within-assay coefficients of variation were 1.3% for TG, 1.9% for cholesterol, 1.1% for LDL, 1.4% for HDL and 0.8% for FBS. Triglyceride value >200 mg/dl, HDL-cholesterol <45 mg/dl, Cholesterol >200 mg/dl, LDL>130 mg/dl were defined as lipid disturbances.

The study was conducted on an outpatient basis according to the principles of the Declaration of Helsinki and was approved by the medical ethics review board of the Endocrinology and Metabolism Research Center (EMRC) of Tehran University of Medical sciences (TUMS). Informed consent was obtained from all volunteers after oral and written information had been given.

The SPSS software version 11.5 and STATA version 8 were used. For statistical analysis the X^2 ^and fisher's exact test were used for comparing the qualitative variables and for quantitative variables with normal distribution we used the parametric tests. Odd's ratio (OR), and 95% confidence interval (95% CI) was used for estimating the effect of shift working on lipid profile, hypertension and high blood glucose levels. Logistic regression modeling was used for multivariable analysis and adjusting the effect of different variables (e.g. age, BMI, eating habits, etc)

## Abbreviations

BP = blood pressure, BMI = body mass index, CHOL = cholesterol, HDL-C=high density lipoprotein-cholesterol, LDL-C = low density lipoprotein-cholesterol, TG = triglyceride

## Authors' contributions

MG, RG participated in the design of the study and performed the data collection

RH, RG performed the statistical analysis, interpretation of data

VH, RH conceived of the study, and participated in its design and coordination

MG drafted the manuscript

VH, AS, PS, SMT revising it critically for important intellectual content

BL final approval of the version to be published

All authors read and approved the final manuscript
